# Summary of the evidence of best practices for the prevention and treatment of embolism syndrome after TACE in primary liver cancer

**DOI:** 10.3389/fonc.2023.1274235

**Published:** 2024-01-15

**Authors:** Yanrong Yao, Xi Huang, Chengsi Zhao, XiaoYan Wang, Guangli Mi, Jingli Liu

**Affiliations:** ^1^ Department of Hepatobilology, General Hospital of Ningxia Medical University, Yinchuan, China; ^2^ School of Nursing, Shanxi University of Chinese Medicine, Xianyang, China; ^3^ Department of Neonatal Intensive Care Unit, General Hospital of Ningxia Medical University, Yinchuan, China; ^4^ School of Nursing, Ningxia Medical University, Yinchuan, China

**Keywords:** primary liver cancer, TACE, embolism syndrome, evidence-based primary liver cancer, evidence-based nursing, best evidence summary

## Abstract

**Objective:**

This study aims to retrieve, evaluate, and summarize domestic and foreign evidence on the prevention and treatment of embolism syndrome following transcatheter arterial chemoembolization (TACE) for primary liver cancer and to provide an evidence-based foundation for clinical practice.

**Methods:**

Utilizing the “6S” model, we conducted a systematic search of UpToDate, BMJ Best Practice, domestic and foreign guidelines, and related databases on the prevention and treatment of embolism syndrome following TACE for primary liver cancer. This search included clinical decision-making, guidelines, systematic reviews, evidence summaries, randomized controlled trials, and expert consensus. The search time frame extended from January 1, 2013 to May 1, 2023. Evidence was synthesized after an independent review of the included studies by two investigators.

**Results:**

A total of 11 articles were included in the analysis, comprising one clinical decision-making article, three clinical guidelines, six expert consensus articles, and one randomized controlled trial. We summarized 31 pieces of evidence across three categories: preoperative preparation, intraoperative interventions, and postoperative symptom management.

**Conclusion:**

This study presents a comprehensive summary of the best available evidence on the prevention and treatment of embolism syndrome following TACE for primary liver cancer. These findings can serve as a valuable reference for clinical practitioners, enabling nurses to deliver individualized care based on the symptoms and specific needs of liver cancer patients.

**Systematic review registration:**

http://ebn.nursing.fudan.edu.cn, identifier ES20232398.

## 1 Introduction

Primary liver cancer (PLC) is a malignant condition that predominantly includes hepatocellular carcinoma (HCC), intrahepatic cholangiocarcinoma (ICC), and combined hepatocellular-cholangiocarcinoma (cHCC-CCA) (1). These cancers are characterized by their insidious onset, rapid progression, and poor prognosis (2). According to the 2020 global cancer statistics report, there were approximately 906,000 new cases of liver cancer, resulting in 830,000 deaths. This malignancy has become the fourth most prevalent malignant tumor worldwide and the second leading cause of cancer-related deaths in China (3, 4). It seriously affects the health of Chinese residents, and the burden of prevention and treatment of liver cancer diseases is increasing.

Primary liver cancer often presents without typical or early-stage symptoms, resulting in missed opportunities for surgical intervention. Surgical resection is feasible for only 10% to 30% of cases, and postoperative recurrence rates are notably high. Despite significant advancements in liver surgery, the majority of newly diagnosed HCC cases are not amenable to surgical resection (5, 6).

With the advancement of interventional medicine, transcatheter arterial chemoembolization (TACE) has emerged as the primary non-surgical palliative treatment for liver cancer patients. TACE involves the injection of embolization agents into tumor blood vessels through the hepatic artery, leading to the blockage of the tumor’s blood supply and inducing ischemic necrosis. This approach serves as a preventive and consolidative measure against recurrence in patients with PLC and has been shown to significantly improve overall patient survival rates (7–9).

While TACE is generally considered a safe procedure, it can lead to various postoperative complications that require caution and consideration. The most common complication is postembolization syndrome (PES), characterized by symptoms such as fever, liver pain, nausea, and vomiting (10). The reported incidence of PES varies widely, ranging from 15% to 90%, which can be attributed to bias arising from differing definitions of embolic syndrome and inadequate follow-up (11). Although PES is typically self-limiting, it significantly affects patient comfort, prolongs hospital stays, and may impact compliance to subsequent treatments (12).

Numerous studies focus on preventing and treating embolism syndrome following TACE, predominantly relying on original research, literature reviews, and meta-analyses. Nonetheless, their conclusions often lack the requisite evidence-based foundation for clinical translation. To address this gap, our study systematically searched both domestic and foreign evidence pertaining to the prevention and treatment of embolism syndrome after TACE and subsequently extracted and summarized this evidence. This study has been registered with the Evidence-Based Nursing Center of Fudan University (ES20232398).

## 2 Materials and methods

### 2.1 Determining the problem and inclusion criteria

Herein the Population, Intervention, Professional, Outcome, Setting, Type of evidence (PIPOST) model was used to determine the basic research questions (13). The inclusion criteria for evidence were as follows: (1) P (population): The evidence’s target population is patients with primary liver cancer after TACE, (2) I (intervention): The measures are aimed at preventing post-TACE syndrome, (3) P (professional): The evidence is applicable to medical staff, patients undergoing TACE, and their family members, (4) O (outcome): The outcome is the incidence of embolism syndrome after TACE, (5) S (setting): The evidence is applicable in medical institutions at all levels, and (6) T (type of evidence): The type of evidence resource include clinical decision-making, best practice, clinical guidelines, systematic reviews, evidence summaries, expert consensus, and relevant original research.

The exclusion criteria were as follows: (1) documents with incomplete data and (2) repeatedly published or updated documents.

### 2.2 Literature search strategy

According to the “6S” model (14) and employing the top-down retrieval principle, we conducted computerized searches in the following evidence-based resource databases: BMJ Best Practice, UpToDate, JBI Library, Cochrane Library, CINAHL, Ontario Registered Nurses Association Guidelines Network, and the National Institute of Health and Clinical Excellence Evidence-based resource databases such as the Guideline Network, the Scottish Intercollegiate Guidelines Network, the New Zealand Guidelines Research Group, the International Guidelines Collaboration Network, the Canadian Medical Association Clinical Practice Guidelines Library, and Medline. A supplementary search of comprehensive databases PubMed, EMBASE, China Biomedical Literature Service System, CNKI, Wanfang database, and VIP database was also carried out. Furthermore, we explored guidelines from reputable organizations, including the European Society of Liver Diseases, the National Comprehensive Cancer Network, the Australian Society of Gastroenterology, the American Society of Liver Diseases and the American Society of Transplantation, the European Society of Nuclear Medicine, International Society for Multidisciplinary Interventional Oncology, Japanese Liver Cancer Research Group, and the European Society for Enhanced Recovery After Surgery. The search period was from January 1, 2013 to May 1, 2023.

We searched for clinical decisions, recommended practices, evidence summaries, clinical practice guidelines, and websites of professional societies. The Chinese search terms included “primary liver cancer/liver tumors/hepatocellular carcinoma/hepatic malignancies”, “percutaneous transarterial chemoembolization/interventional therapy for liver cancer”, “treatment/hepatic arterial embolization/hepatic arterial chemoembolization”, and “post-embolization syndrome/embolism syndrome/abdominal pain/fever/nausea/vomiting”. The English search terms included “primary liver cancer/liver neoplasms/hepatocellular carcinoma”, “transarterial chemoembolization/transfemoral artery intervention/hepatic chemoembolization/chemoembolization/hepatic artery * embolization/embolization”, and “postembolization/postembolization syndrome/pain/nausea/vomiting/fever”.

The Chinese search strategy using CNKI as an example was as follows: SU % = (‘primary liver cancer’ + ‘liver cancer’ + ‘primary liver tumor’ + ‘primary liver cancer’ + ‘hepatocellular carcinoma’ + ‘cholangiocarcinoma’ + ‘intrahepatic cholangiocarcinoma’ + ‘hepatic angiosarcoma’ + ‘hepatoblastoma’ + ‘TACE’ + ‘percutaneous hepatic arterial chemoembolization’ + ‘transarterial chemoembolization for hepatocellular carcinoma’ + ‘interventional therapy for liver cancer’ + ‘hepatic arterial embolization’ + ‘hepatic arterial chemoembolization’) * (‘post-embolism syndrome’ + ‘embolism syndrome’ + ‘PES’ + ‘abdominal pain’ + ‘nausea’ + ‘vomiting’ + ‘fever’ + ‘pain in the liver area’ + ‘abdominal distension’ + ‘anorexia’) * (‘treatment’ + ‘prevention’ + ‘management’ + ‘diagnosis and treatment’ + ‘nursing’) * (‘guidelines’ + ‘meta’ + ‘systematic review’ + ‘evidence summary’ + ‘practice guideline’ + ‘expert consensus’ + ‘randomized control’ + ‘RCT’).

The English search strategy using PubMed as an example was as follows:

1 (TACE [Title/Abstract] OR transcatheter arterial chemoembolization [Title/Abstract] OR transfemoral artery intervention [Title/Abstract] OR transcatheter hepatic arterial chemoembolization [Title/Abstract] OR transcatheter hepatic arterial chemoembolization [Title/Abstract] OR percutaneous hepatic artery chemoembolization [Title/Abstract] OR trans-arterial chemoembolization [Title/Abstract] OR percutaneous transcatheter arterial chemoembolization [Title/Abstract] OR transarterial chemo-embolization [Title/Abstract]), #2 (embolization syndrome [Title/Abstract] OR embolism syndrome [Title/Abstract] OR embolic syndrome [Title/Abstract] OR post-embolization syndrome [Title/Abstract] OR embolism syndrome after interventional therapy [Title/Abstract] OR PES [Title/Abstract]), #3 (systematic review [Title/Abstract] OR meta-analysis [Title/Abstract] OR guideline [Title/Abstract] OR evidence summary * [Title/Abstract] OR consensus [Title/Abstract] OR RCT * [Title/Abstract]), and #4 (#1 AND #2 AND #3 AND Filters: from 2013 to 2023).

### 2.3 Literature quality evaluation

For guideline evaluation, we employed the British “Clinical Guidelines Research and Evaluation System” (AGREE II) (15) for quality evaluation, including 23 items in six areas: scope and purpose, participants, rigor of formulation, clarity, applicability, and editorial independence. Each item was rated on a scale of 1 to 7, with 7 indicating full compliance and 1 indicating non-compliance. For expert consensus evaluation, we followed the JBI Evidence-Based Health Care Center Expert Consensus (2017) evaluation standard (16) for quality assessment. Randomized controlled trials (RCTs) were evaluated using the JBI Evidence-Based Health Care Center’s RCT paper quality evaluation tool (17).

### 2.4 Summary, classification, and recommendation level of evidence

The quality of evidence and the level of recommendation were assessed independently by two researchers. In cases of conflicting assessments, a third researcher participated in discussions to reach a consensus. When discrepancies arose among evidence from different sources, we applied principles prioritizing evidence-based, high-quality, and recently published evidence.

The clinical practice guidelines and clinical decisions included were graded using their original grading system, while evidence lacking a grading system was graded using the JBI Evidence Pre-grading System (2014 version) to grade the original study on a scale of 1–5, with level 1 being the highest and level 5 being the lowest.

The recommendation level of evidence adopts the JBI Evidence Pre-grading and Evidence Recommendation Level System (2014 version) and refers to the grading system provided by the evidence itself.

## 3 Results

### 3.1 Literature search results

We initially retrieved a total of 1,057 documents through a systematic search. After removing duplicates using NoteExpress and applying our inclusion criteria, we obtained the full-text copies of potentially relevant documents. Each full-text copy was carefully reviewed to confirm whether it met the inclusion criteria. Eventually, we included a total of 11 documents, comprising one clinical decision (18), three guidelines (7, 19, 20), six expert consensus (21–26), and one randomized controlled trial (27). The details of the included literature are summarized in **Table 1**.

**Table 1:** Basic characteristics of the included literature (*n* = 11).

Included literature	Topic of literature	Source	Year	Country	Type of literature
Steven A. Curley (18)	Localized hepatocellular carcinoma: liver-targeted therapy for patients ineligible for surgery and ineligible for localized thermal ablation	UpToDate	2022	USA	Clinical decision
National Health Commission of the People’s Republic of China Medical Administration (19)	Primary liver cancer diagnosis and treatment guidelines	Medline	2022	China	Guideline
Chinese Medical Doctor Association interventional physician branch clinical guidelines committee (7)	Clinical practice guidelines for transarterial chemoembolization of hepatocellular carcinoma in China	Medline	2021	China	Guideline
Paul J. Hesketh (20)	Antiemetic measures of radiotherapy and chemotherapy in cancer patients	ASCO	2020	USA	Guideline
2019 Expert Committee of Liver Cancer Clinical Collaboration between Chinese and Western Medicine (21)	Expert consensus on interventional diagnosis and treatment of primary liver cancer	Medline	2021	China	Expert consensus
Multidisciplinary Collaborative Group of Digestive Tract Tumors, Cancer Hospital of Peking Union Medical College, Chinese Academy of Medical Sciences (22)	Expert guidance on Internet management of common drug treatment-related adverse reactions in patients with liver cancer	CNKI	2021	China	Expert consensus
Interventional perioperative special Committee of Interventional Physician Branch of Chinese Medical Doctor Association (23)	Expert consensus on perioperative pain management in interventional treatment of liver malignancies	CNKI	2022	China	Expert consensus
Research group of the National Science and Technology Special Project for the prevention and control of major infectious diseases such as AIDS and viral hepatitis (24)	Expert consensus on the rehabilitation of traditional Chinese and Western medicine after transcatheter arterial chemoembolization for primary liver cancer	CNKI	2021	China	Expert consensus
Chinese Anticancer Association tumor clinical chemotherapy professional committee (25)	Expert consensus on prevention and treatment of nausea and vomiting related to cancer drug therapy in China	Medline	2022	China	Expert consensus
Nursing Group, Interventional Medicine Branch of Hubei Medical Association (26)	Expert consensus on perioperative nursing strategy of transcatheter arterial chemoembolization for hepatocellular carcinoma	CNKI	2022	China	Expert consensus
Sadahisa Ogasawara (27)	Randomized placebo-controlled trial of prophylactic dexamethasone for transcatheter arterial chemoembolization	Cochrane Library	2018	China	RCT

ASCO, American Society of Clinical Oncology.

### 3.2 Quality evaluation results of the included literature

#### 3.2.1 Quality evaluation results of the guideline

In this study, we included three guidelines (7, 19, 20), and the quality evaluation results are detailed in **Table 2**.

**Table 2:** Quality evaluation results of the guidelines (*n* = 3).

Included literature	Percentage of standardization by field (%)						Number of fields ≥60%	Number of fields ≥30%	Recommendation level
	Scope and purpose	Participant	Preciseness	Clearness	Applicability	Independence			
National Health Commission of the People’s Republic of China Medical Administration (19)	94.50	83.33	71.43	78.57	66.08	100	6	6	A
Chinese Medical Doctor Association Interventional Physician Branch Clinical Guidelines Committee (7)	97.62	85.71	69.64	97.62	67.86	89.29	6	6	A
Paul J. Hesketh (20)	100	78.58	78.57	92.86	58.93	89.29	5	6	B

A, reliable evidence of effectiveness with clear clinical benefits, strongly recommended. B, may produce reliable or moderate evidence of effectiveness with limited clinical benefit, General recommendation.

#### 3.2.2 Quality evaluation results of the expert consensus

Six expert consensus articles were included in this study (21–26), and the quality evaluation results are shown in **Table 3**.

**Table 3:** Quality evaluation results of expert consensus (*n* = 6).

Included literature	①	②	③	④	⑤	⑥	Whether to include
2019 Expert Committee of Liver Cancer Clinical Collaboration between Chinese and Western medicine (21)	Y	Y	Y	Y	Y	Y	Y
Multidisciplinary Collaborative Group of Digestive Tract Tumors, Cancer Hospital of Peking Union Medical College, Chinese Academy of Medical Sciences (22)	Y	Y	Y	Y	Y	Y	Y
Interventional Perioperative Special Committee of Interventional Physician Branch of Chinese Medical Doctor Association (23)	Y	Y	Y	Y	Y	Y	Y
Research Group of the National Science and Technology Special Project for the prevention and control of major infectious diseases such as AIDS and viral hepatitis (24)	Y	Y	Y	Y	Y	Y	Y
Chinese Anticancer Association Tumor Clinical Chemotherapy Professional Committee (25)	Y	Y	Y	Y	Y	Y	Y
Nursing Group, Interventional Medicine Branch of Hubei Medical Association (26)	Y	Y	Y	Y	Y	Y	Y

①Are the sources of the ideas clearly marked? (YES/NO) ②Does the opinion come from an influential expert in the field? (YES/NO) ③Whether the point of view presented is centered on the relevant population interests of the study? (YES/NO) ④Are the stated conclusions based on the analysis? Are ideas expressed logically? (YES/NO)⑤Was there any reference to other literature? (YES/NO) ⑥Are there any inconsistencies between the ideas presented and the previous literature? (YES/NO). Letter Y, indicates whether criteria ①-⑥ are met and decides whether to include evidence.

#### 3.2.3 Quality evaluation results of clinical decision-making

One clinical decision (18) from UpToDate was directly included.

#### 3.2.4 Quality evaluation results of randomized controlled trials

A randomized controlled trial (27) from the Cochrane Library was included, and its quality was evaluated using evaluation tools. The evaluation result of item 6, “Whether the results evaluator was blinded,” was “unclear”, and the evaluation result of the other item was “Yes”. The overall quality was high.

### 3.3 Evidence description and summary

**Figure 1:**
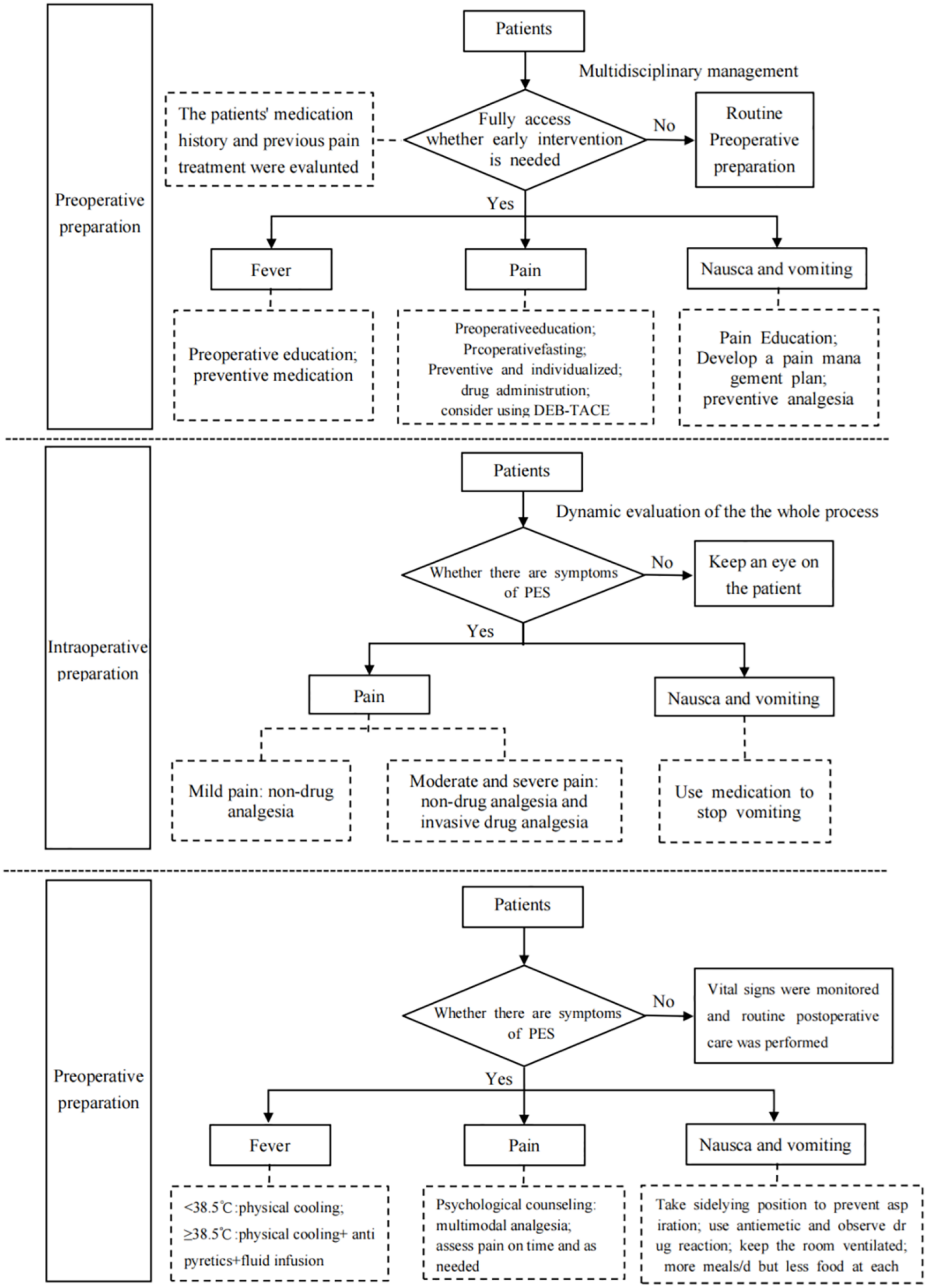
Flow chart of evidence transformation for prevention and treatment of embolic syndrome after transcatheter arterial chemoembolization.

According to the principle of evidence synthesis, the researchers summarized the evidence. A total of 31 pieces of evidence were summarized, divided into 3 evidence topics, as shown in **Table 4** (preoperative preparation, intraoperative interventions, and postoperative symptom management). A flow chart of evidence transformation for the prevention and treatment of embolism syndrome after TACE is shown in **Figure 1**.

**Table 4:** Summary of the best evidence for the prevention and treatment of embolism post-TACE syndrome for primary liver cancer.

Category of evidence	Evidence description	Source	Evidence level	Recommendation level
Preoperative preparation				
Multidisciplinary management	1. Establish a comprehensive Internet management platform or follow-up system to manage drug treatment-related adverse reactions in liver cancer patients, including doctors, medical assistants, health managers, nutritionists, and psychological counselors (22, 25)	Expert consensus	5	A
	2. Refer patients with liver cancer to specialized centers with multidisciplinary expertise for comprehensive assessment, monitoring, and treatment, considering all available treatment options (18)	One quasi-experimental study and one retrospective cohort study	2	A
	3. Implement multimodal analgesia principles by establishing a multidisciplinary pain management team that includes anesthesia, intervention, and nursing. This team should conduct comprehensive pain assessments and provide continuous, safe, and effective pain management to enhance patient comfort and satisfaction (23)	Expert consensus	5	A
Fever	4. Educate patients about the cause of fever and pain, which is often related to local tissue ischemia and necrosis after hepatic artery embolization (19, 24)	Guidelines and expert consensus	5	B
	5. Prophylactic use of corticosteroids and 5‐HT3 receptor antagonists to effectively reduce fever after TACE (27)	A randomized controlled trial	1	B
Pain	6. Conduct a comprehensive assessment within 8 h of admission, including the patient’s physical and psychological state, chronic pain history, previous pain treatments, medication history, etc. (23, 26)	Expert consensus	5	B
	7. Evaluate the patient’s surgical tolerance before surgery or on the day of surgery and develop an intraoperative pain treatment plan to enhance patient tolerance and experience (23, 26)	Expert consensus	5	B
	8. Programmed and personalized pain education before surgery to increase patients’ awareness of pain, reduce fear, and ensure appropriate analgesia (23)	Three randomized controlled trials	1	A
	9. Preoperative preventive analgesia for patients with chronic pain, opioid tolerance, etc. (26)	Expert consensus	5	B
Nausea and vomiting	10. Recommend TACE with drug-eluting beads (DEB-TACE) over traditional TACE therapy, as it reduces systemic exposure to chemotherapy (18)	A randomized controlled trial	1	B
	11. Advise fasting for 4–6 h before surgery and avoiding contact with cooking or eating to reduce irritation (7, 22)	Guidelines and expert consensus	5	A
	12. Choose an antiemetic regimen based on the emetic risk of the antineoplastic drug, evaluate high-risk factors and concomitant diseases, and provide preventive antiemetic drugs before treatment (22, 25)	Expert consensus	5	A
	13. Administer preventive antiemetic medication before each antitumor treatment (intravenous injections 30 min before the first dose, oral preparations 60 min before the first dose, and granisetron transdermal patches applied 24–48 h before the first dose) to cover the entire risk period (20, 25)	Guidelines and expert consensus	2	B
	14. Prophylactic use of corticosteroids and 5‐HT3 receptor antagonists to effectively reduce nausea and vomiting (18, 27)	Clinical decisions and a randomized controlled trial	1	B
	15. Predict or assess the possibility of nausea and vomiting based on the patient’s previous experiences. Adjust the antiemetic regimen for the next cycle of antineoplastic treatment based on the efficacy observed in previous cycles (22, 25)	Expert consensus	1	A
Intraoperative interventions				
Pain	16. Dynamic monitoring of the patient’s vital signs during the entire surgical procedure to ensure patient safety (23, 26)	Expert consensus	5	A
	17. For mild pain, we recommend non-drug analgesia nursing, especially psychological nursing, pay full attention to the pain-inducing effect of psychological factors, take the initiative to care patients, relieve their tension and fear, and inform the operation process in time, teaching patients psychological adjustment methods (23)	A cohort study	2	B
	18. For moderate and severe pain, it is recommended to give invasive drug analgesia on the basis of non-drug analgesia nursing (23)	A quasi-experimental study	1	A
	19. Address intraoperative pain promptly to avoid increased blood pressure and irritability, which could pose risks during surgery. Administer appropriate painkillers as directed by the doctor, such as non-steroidal drugs like flurbiprofen, local anesthetics like lidocaine, or opioids like morphine, to ensure the smooth progression of the operation (23, 26)	A systematic review of several experimental studies	2	A
Nausea and vomiting	20. Routinely use gastric mucosal protectants and dexamethasone to manage nausea and vomiting reactions (20, 25)	Guidelines and expert consensus	2	B
Postoperative symptom management				
Fever	21. Would recommend simplifying: manage fever by providing physical cooling, administering antipyretic drugs if necessary, and ensuring adequate fluid replacement (18, 24, 26)	Clinical decision and expert consensus	2	A
	22. Change underwear and bed sheets promptly if there is excessive sweating to maintain skin dryness and comfort. Encourage patients to stay hydrated to prevent dehydration (24)	Expert consensus	5	B
Pain	23. Provide psychological counseling to patients and use various methods like empathy, music, relaxation, and distraction to help manage pain. Consider three-step analgesic therapy for cancer pain if the pain persists (7, 19, 23, 24, 26)	Guidelines and expert consensus	3	B
	24. Evaluate the intervention’s effect and any related adverse reactions after surgery. Develop a pain management plan and follow-up plan before discharge (23, 26)	Expert consensus	5	B
	25. Observe the nature and location of abdominal distension and pain and check for any signs of acute abdomen in the upper abdomen to identify liver rupture (26)	Expert consensus	5	B
	26. Evaluate pain every 2 h post-surgery using appropriate pain assessment methods (e.g., numerical grading, visual analog scale, or facial expression assessment). Administer non-drug or drug-based analgesics based on pain scores. If the patient’s pain score is between 1 and 3 points, weak analgesics should be used to control pain; if the patient’s pain score is ≥4 points, use non-steroidal anti-inflammatory drugs and opioids to control the pain; if the pain score is ≥4 points, use an opioid drug and consider using an intravenous analgesic pump for delivery (23, 26)	A quasi-experimental study and expert consensus	1	A
Nausea and vomiting	27. If vomiting occurs, place the patient on his or her side, turn the head to one side to facilitate vomit removal, and avoid aspiration. Provide oral hygiene care (22)	Expert consensus	5	A
	28. Observe the nature, color, and volume of vomit to detect signs of gastrointestinal bleeding. Administer antiemetics as prescribed and monitor the patient’s response and electrolyte balance (24, 26)	A quasi-experimental study and expert consensus	2	A
	29. Eliminate irritating odors in the room and maintain appropriate lighting as well as ventilation (22, 24)	A quasi-experimental study and expert consensus	2	A
	30. Follow dietary principles such as small and frequent meals, choosing easily digestible foods, avoiding spicy or irritating foods, and not consuming excessively hot or cold foods. Consider using acidic fruits to relieve nausea (22)	Expert consensus	5	B
	31. If vomiting is frequent, fast for 4 to 8 h, then gradually introduce a liquid diet. Transition to a refined protein, high-vitamin, high-carbohydrate, and low-fat diet as appropriate after 2 days. Enhance the appeal of food through color, aroma, and taste and encourage small, frequent meals. Consider adding ginger to the diet for nausea relief. Enteral or parenteral nutritional support may be needed for patients with severe dietary disturbances (24)	Expert consensus	5	B

A, reliable evidence of effectiveness with clear clinical benefits, strongly recommended. B, may produce reliable or moderate evidence of effectiveness with limited clinical benefit, General recommendation.

## 4 Discussion

### 4.1 Clinical significance of the prevention and treatment of embolism syndrome after TACE based on evidence summary

TACE is an established treatment option for patients with unresectable liver disease, prolonging survival and improving the quality of life (28). Embolism syndrome, or at least some characteristic symptoms, is almost unavoidable following TACE (11). The predictive model constructed by Meredith C. Mason indicated that the bilobal and single-lobe distribution of tumors and the use of DEB-TACE were independent predictors of PES (11). The study of Mohamed H. Khalaf also pointed out that PES history, tumor burden, and DEB-TACE were important predictors of prolonged recovery (29). According to the study of Natascha Roehlen, preoperative tumor size, degree of cirrhosis, and DEB-TACE are predictors of PES (30). Embolism syndrome not only greatly impacts patient comfort post-treatment but also frequently leads to protracted hospital stays and treatment progression. Moreover, it emerges as a complicating factor in the diagnostic and therapeutic processes for healthcare practitioners, potentially giving rise to diagnostic complexities in cases characterized by fever and pain, thereby necessitating supplementary diagnostic tests (31). Effective management of embolic syndrome is important from a clinical practice point of view, but there are currently no established guidelines. Although UpToDate and several countries have published guidelines for the diagnosis and treatment of liver cancer or clinical decision-making information, the evidence is long and the evidence for the prevention and treatment of embolic syndrome after TACE is relatively scattered. Therefore, following a rigorous process of quality assessment and evidence grading, this study aims to consolidate the most robust evidence pertaining to the prevention and treatment of embolism syndrome in patients undergoing TACE.

This summary of evidence fully focuses on the evidence related to the prevention and treatment of embolic syndrome after TACE surgery, which will help nursing practitioners to acquire and understand the evidence efficiently, establish standardized prevention and management procedures for embolic syndrome after TACE, improve nursing practitioners’ sensitivity to PES, reduce the incidence of PES, and reduce patient hospitalization time and cost.

### 4.2 Forming a multidisciplinary team and an Internet platform to strengthen the health education and follow-up system

Embolism syndrome after TACE is an unavoidable clinical problem marked by its multifaceted symptoms. Formulating and implementing effective and precise prevention and treatment strategies necessitate the collaborative efforts of a multidisciplinary team. This team typically comprises oncologists, nurses specializing in oncology care, gastroenterologists, pain management specialists and nurses, psychological counselors, nutritionists, and other healthcare professionals. Within this framework, physicians shoulder the responsibilities of symptom assessment and the development of tailored management plans, while nurses oversee patients, ensuring regular monitoring and recording of patient data. Nutritionists and psychological counselors, on the other hand, are entrusted with personalized management strategies tailored to the unique needs of individual patients. Patients with liver cancer have recurrent symptoms, and continuous management outside the hospital is an important part of systemic treatment. Establishing an Internet-based management platform for follow-up care is invaluable for healthcare managers. This platform streamlines the identification of symptoms and the monitoring of critical health indicators, enabling timely treatment recommendations. Beyond its utility in facilitating multidisciplinary team collaboration, the Internet management platform also extends practical guidance to liver cancer patients as they navigate the complexities of systemic treatment outside the hospital setting to maximize the benefits of treatment for these patients (22).

### 4.3 Strengthening the evaluation of each node is an important measure to prevent and treat embolism syndrome

Effective symptom management in surgical care relies on timely and methodical symptom assessment. Prior to surgery, there is a need to promote robust public education by introducing patients to the surgery’s purpose, process, and benefits. This builds patient confidence and provides essential psychological support (32). During surgery, treatment plans to address adverse reactions are implemented, improving patient tolerance and experience. Continuously monitoring patient complaints, maintaining open communication, and promptly identifying and addressing issues also enhance surgical safety. The patients are monitored post-surgery for adverse reactions and discomfort, offering empathetic responses to their concerns. To boost patient tolerance and treatment compliance before discharge, thorough assessments are conducted, psychological support is provided, and follow-up plans are developed.

### 4.4 Limitations of this study

This study has certain limitations. Notably, some systematic reviews and meta-analyses were excluded during our systematic search as they focused on survival time or tumor response as outcome measures. Additionally, several studies comparing various TACE techniques (cTACE or DEB-TACE), combinations of TACE with other treatments, and different drug predictions were omitted due to incomplete study designs or low-quality assessments. At the same time, the literatures included in this study were mainly from Asia. The included people had different concepts and attitudes toward PES, and there were regional and cultural differences caused by different medical service systems and medical environments. Therefore, future research should aim to incorporate high-quality original studies to gain deeper insights into the effects of various TACE techniques and drug dosages on embolism syndrome.

## 5 Summary

This study summarized 31 pieces of evidence for preventing and treating embolism syndrome in patients after TACE, serving as a valuable resource for clinical medical practitioners. The results presented here are derived from existing research conclusions. Nevertheless, individual patient cases may vary, necessitating tailored treatment plans based on specific patient conditions. When incorporating this evidence into clinical practice, healthcare professionals should exercise their expertise and consider the unique clinical circumstances, using the best available evidence as a foundation for informed decision-making in clinical nursing practice.

## Data availability statement

The datasets generated and/or analyzed during the current study are available from the corresponding author on reasonable request. Requests to access these datasets should be directed to yaoyanrong110@163.com.

## Author contributions

YY: Funding acquisition, Writing – original draft, Writing – review & editing, Data curation, Methodology, Project administration. XH: Data curation, Formal analysis, Writing – original draft. CZ: Methodology, Writing – review & editing. XW: Formal analysis, Writing – review & editing. GM: Conceptualization, Resources, Writing – review & editing. JL: Project administration, Resources, Writing – review & editing.
